# Transtibial repair of medial meniscus posterior root tears: Interference screw fixation improves primary stability in a porcine model

**DOI:** 10.1002/jeo2.70488

**Published:** 2025-11-03

**Authors:** Timo Spannagel, Bastian Schäfer, Philip Anderson, Isabell Biermann, Stephan Reppenhagen

**Affiliations:** ^1^ Department for Orthopedic Surgery, Koenig‐Ludwig‐Haus University of Wuerzburg Wuerzburg Germany; ^2^ Department of Orthopedic and Trauma Surgery, Juliusspital Klinikum Wuerzburg Mitte Wuerzburg Germany; ^3^ Department of Functional Materials in Medicine and Dentistry University of Wuerzburg Wuerzburg Germany

**Keywords:** interference screw, meniscus posterior root, root tear, root tear refixation, transtibial pull‐out

## Abstract

**Purpose:**

This porcine study aimed to evaluate surgical refixation methods for medial meniscal root tears. We compared biomechanical characteristics of tibial fixation using an interference screw (IS) and fixation with an Endobutton (EB).

**Methods:**

Forty porcine knee joints were prepared with radial section for a complete detachment of the medial meniscus posterior root (MMPR), followed by transtibial pull‐out refixation. Specimens were randomly assigned to four groups based on fixation method and suture material: IS (STORZ PEEK Power Fix 7 × 25 mm, Storz, Tuttlingen, Germany) or EB (Smith & Nephew, London, UK), each combined with two single stitch sutures of either FiberWire size 2 (FW; Arthrex, Naples, USA) or SutureTape 1.3 mm (ST; Arthrex, Naples, USA). Constructs were subjected to cyclic loading (1000 cycles), followed by load‐to‐failure testing. Biomechanical parameters assessed included elongation, stiffness, yield load, maximum load to failure and failure mode.

**Results:**

Fixation with IS (Groups 1 and 2) demonstrated significantly lower elongation and greater stiffness compared to EB fixation (Groups 3 and 4). Specifically, Group 2 (IS + FW) showed significantly reduced elongation versus both EB groups (*p* < 0.05). Group 1 (IS + ST) exhibited significantly higher stiffness than Groups 3 and 4 (*p* < 0.05). Yield load and maximum load to failure varied depending on the fixation method and suture type. Group 4 (EB + FW) had the highest yield load, followed by Group 1 (IS + ST) and Group 3 (EB + ST). Group 2 (IS + FW) had a significantly lower yield load compared to Group 4 (*p* < 0.05). Furthermore, Group 2 demonstrated significantly reduced maximum load to failure compared to both Group 1 and Group 4 (*p* < 0.05). Overall, Group 1 (IS + ST) achieved the highest maximum load to failure, whereas Group 2 (IS + FW) showed the lowest.

**Conclusion:**

In this porcine model, transtibial pull‐out repair of MMPR tears using IS demonstrated superior primary biomechanical stability compared to EB fixation. Constructs with IS showed reduced elongation and increased stiffness under load. However, to confirm the clinical relevance of these findings, further investigations in clinical settings are necessary.

**Level of Evidence:**

N/A.

**Abbreviations**
ANOVA: analysis of variance
D‐DLL: double double‐locking loop
EB: Endobutton
FW: FiberWire
IS: interference screw
MK: modified Kessler
MMA: modified Mason–Allen
MMPR: medial meniscus posterior root
MMPRT: medial meniscus posterior root tear
ST: SutureTape
S‐DLL: single double‐locking loop
TSS: two simple sutures
(M)ME: (medial) meniscus extrusion

## BACKGROUND

Posterior root tears of the medial meniscus (MMPRTs), defined as tears or avulsions occurring within 9 mm of the bony root attachment, account for approximately 10.1%–27.8% of all meniscal tears [3, 19, 31, 47]. The meniscal roots are critical for transmitting axial loads and maintaining hoop stress during knee flexion [14, 40], with the posterior part of the meniscus bearing the highest loads in flexion [54]. A radial MMPRT disrupts circumferential fibre integrity [39] and biomechanically resembles a total medial meniscectomy [1], leading to altered joint kinematics [39], increased peak contact pressures [1], medial meniscal extrusion (MME) [12, 21, 23] and accelerated cartilage degeneration [16, 37]. Furthermore, MMPRTs have been associated with osteonecrosis [49, 57], as well as progression of osteoarthritis [2] due to the resulting increase in medial compartment mobility and tangential shear forces following disruption of the posterior root structure [39, 59].

Considering these consequences, surgical refixation is currently the recommended treatment [44]. MMPRT repair can improve tibiofemoral contact mechanics [24], restore hoop tension and joint pressure distribution [27, 35, 40, 48] and may delay osteoarthritic progression [60]. Anatomical reattachment is essential [30, 55] and is typically achieved using either suture anchor techniques [22, 26] or transtibial pullout repair [7, 17, 18, 27, 28, 34, 43, 53]. While suture anchor repair has shown less displacement and higher stiffness in some biomechanical comparisons [10], findings remain inconsistent [61] and both techniques appear clinically equivalent [25]. Nevertheless, transtibial pullout repair remains the most widely adopted approach [20], despite known challenges such as tunnel‐related suture elongation and micromotion [10, 51], tibial fixation loosening and displacement at the meniscus‐suture interface [5]. Further refinement of this technique is therefore warranted [33].

To date, biomechanical studies have largely evaluated tibial fixation techniques [61], suturing methods [32] or suture materials [11, 41] in isolation. However, the interaction between these components may critically influence overall repair stability. Since mechanical failure can occur at the tibial fixation site, along the suture path, or at the meniscus‐suture interface, a comprehensive assessment of full repair constructs is necessary. Interference screw (IS) fixation may reduce micromotion by shortening the free suture length within the tunnel, while FiberWire (FW) offers low intrinsic elongation [41] but may fail by cutting through meniscal tissue or passing by the screw under low load. Optimising the combination of fixation techniques and materials is crucial to minimise cyclic elongation, increase stiffness, and achieve high primary pullout strength. These parameters are key determinants of successful meniscal healing throughout the early postoperative rehabilitation period [32, 45, 56]. Reduced elongation and higher construct stiffness may, in the best case, contribute to limiting MME and sufficient coverage of the posterior tibial plateau [36], which is strongly associated with cartilage degeneration, bone marrow lesions, and osteoarthritis progression [38, 62]. As limiting MME is one of the primary goals of MMPRT repair [6], biomechanical optimisation is of high relevance.

In this study, we employed a porcine model because it closely resembles the human knee in terms of healing potential, anatomical structure, vascular supply, and meniscal weight [8] so it represents a relevant and widely accepted model for investigating the biomechanical behaviour of meniscal repair techniques [9, 13, 51, 61].

We hypothesised that tibial fixation using IS is biomechanically superior to fixation using Endobutton (EB). This biomechanical superiority of IS is likely dependent on the suture material it is combined with.

## MATERIALS AND METHODS

### Specimens

A total of 40 freshly frozen porcine knees, including their medial menisci, were utilised to perform a repair using transtibial pullout techniques with two different tibial fixations and two different suture materials.

The porcine knees were all sourced from the same abattoir, ensuring comparable conditions for growing up and testing. They were slaughtered at an age of 6 months on the day before testing and subsequently shock‐frozen at −40°C. On the day of testing, they were slowly thawed at room temperature for 10 h. Careful dissection was performed to expose the medial meniscus without causing further damage. Special attention was paid to ensuring that the medial menisci were macroscopically free from degeneration and that the medial meniscus posterior roots (MMPRs) and cartilage were intact. The menisci were then kept moist with isotonic saline solution.

### Testing conditions

**Figure 1:**
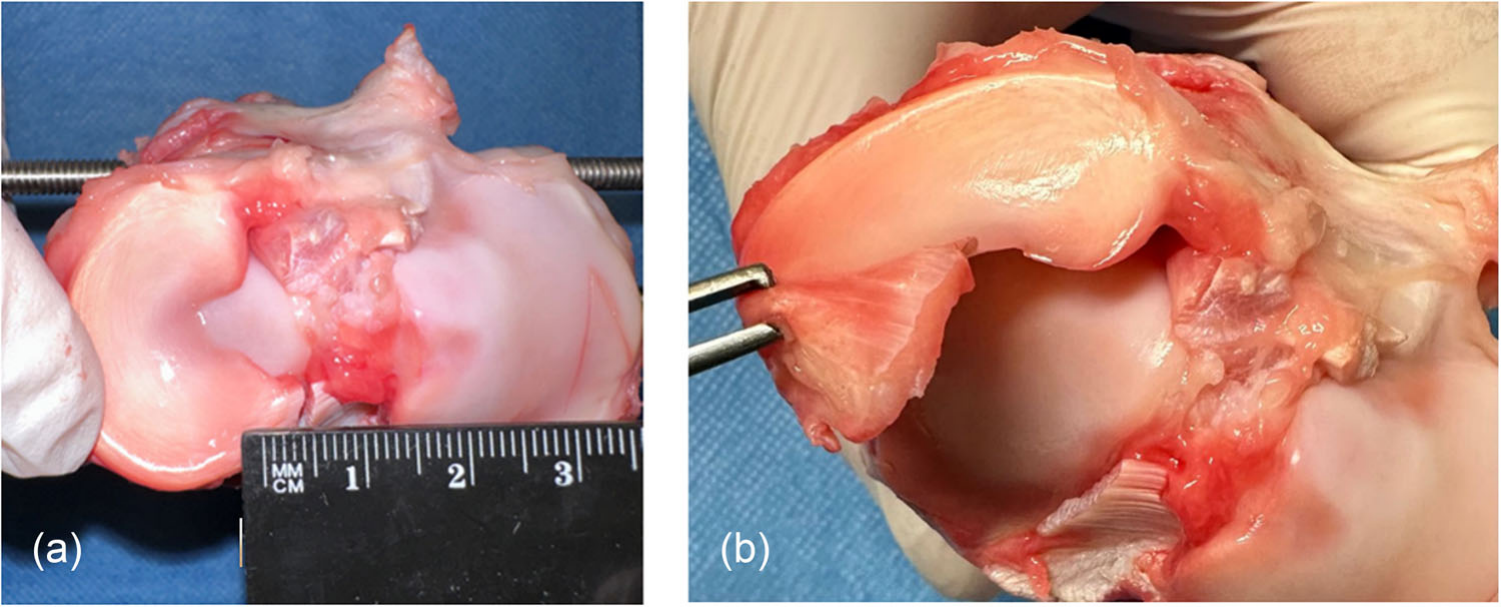
Preparation of the medial meniscus posterior root. (a) The meniscal root was sharply cut with a scalpel 5 mm medial to its posterior tibial insertion, similar to a radial tear (b) including the meniscotibial ligament for a complete detachment.

The knees were randomised into Groups 1–4: IS and ST (Group 1), IS and FW (Group 2), EB and ST (Group 3), and EB and FW (Group 4). In each group, 10 MMPR‐repairs were tested. In all groups, the meniscal root was sharply cut 5 mm medial to its posterior tibial insertion with a scalpel, similar to a radial tear including the meniscotibial ligament for a complete detachment (Figure 1a,b).

### Repair technique

Next, the drill channel for suture shuttling was prepared. Using an ACL targeting device (Arthrex, Naples, USA), a *K*‐wire was first drilled at a 60° angle from the medial side of the proximal tibia to the dorsal tibial plateau. The exit point of the wire was located 6 mm anterior to the posterior edge of the medial tibial plateau and 10 mm medial to the bony attachment of the MMPR. After positioning the wire, it was overdrilled with a 4.5 mm cannulated drill.

**Figure 2:**
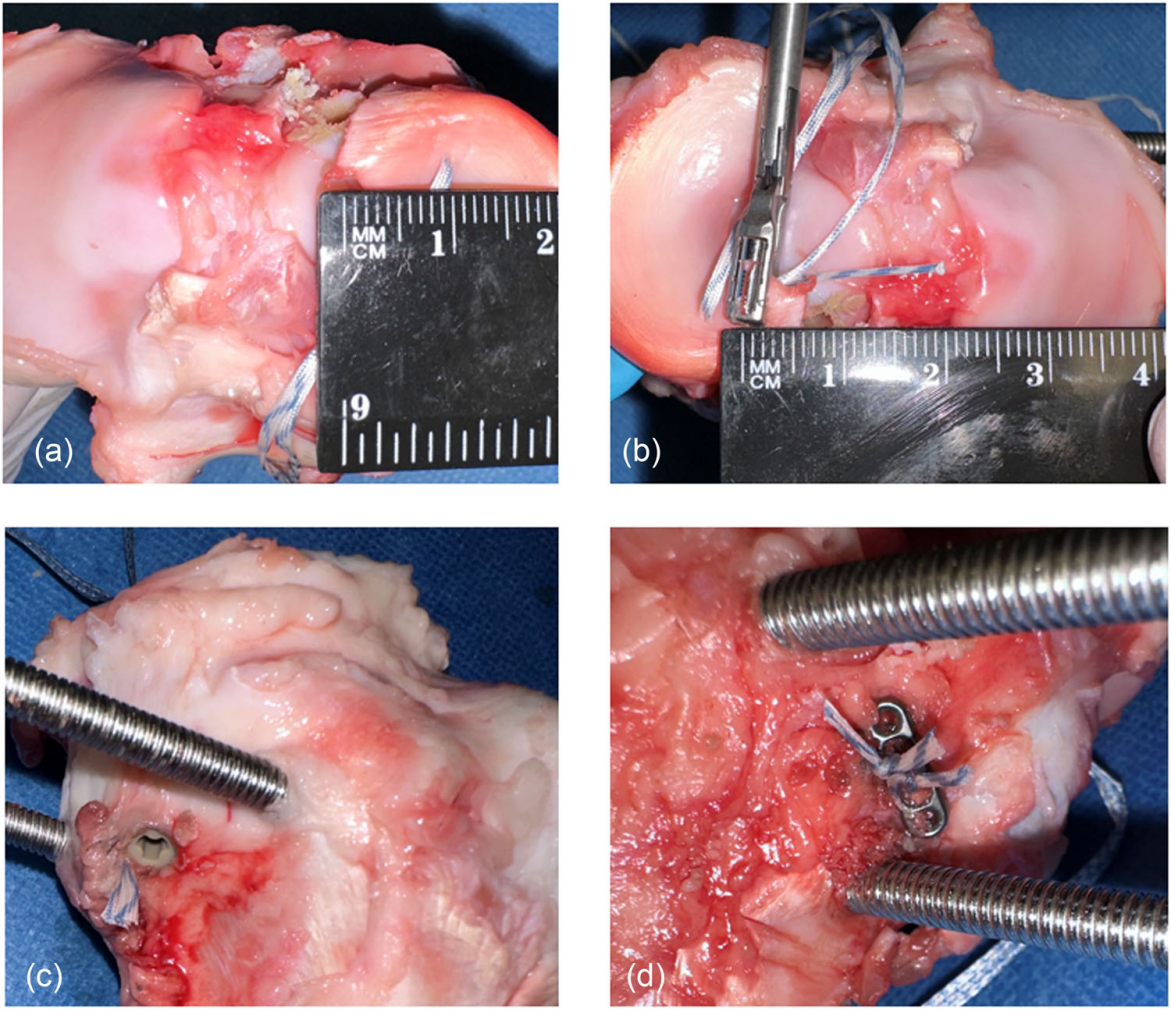
Sutering of the medial meniscus posterior root and tibial fixation of the sutures. (a) The medial suture was placed 9 mm medial to the tear. (b) The lateral suture was placed 6 mm medial the tear. Then the sutures were fixed tibially with (c) an interference screw (STORZ PEEK Power Fix 7 × 25 mm) or (d) an Endobutton.

For the suturing technique, two single‐stitch sutures perpendicular to the fibre direction of the meniscus were placed in all groups. Depending on the test group, either two 1.3 mm SutureTape (Arthrex, Naples, USA) or two FiberWire size 2 (Arthrex, Naples, USA) were used. These were positioned so that the medial suture was 9 mm medial to the tear and the lateral one was 6 mm (Figure 2a,b). The stitches were placed using FIRSTPASS MINI (Smith & Nephew, London, UK) in a manner that encompassed at least more than half of the meniscal width from central to peripheral. Finally, the sutures were shuttled through the drill channel and fixed transtibially as follows.

#### Interference screw

A 7 × 25 mm non‐bioresorbable IS (STORZ PEEK Power Fix 7 × 25 mm; Storz, Tuttlingen, Germany) tibially fixed the sutures (Figure 2c). These were tensioned solid to ensure precise re‐adaptation of the MMPR, and the sutures were then firmly blocked with the IS by hand. Subsequently, the tibially protruding suture material was cut with a scalpel to a length of 4 mm. The IS flushly closed with the tibial cortex.

##### Endobutton

Each of the suture ends were pulled through the central holes of an EB (Smith & Nephew, London, UK). Then the EB was placed flush on the anterior edge of the tibia. The sutures were tensioned so that the MMPR adapted smoothly. The paired suture ends were knotted on the EB with four knots each. The remaining suture ends were cut with a scalpel to a length of 4 mm (Figure 2d).

**Figure 3:**
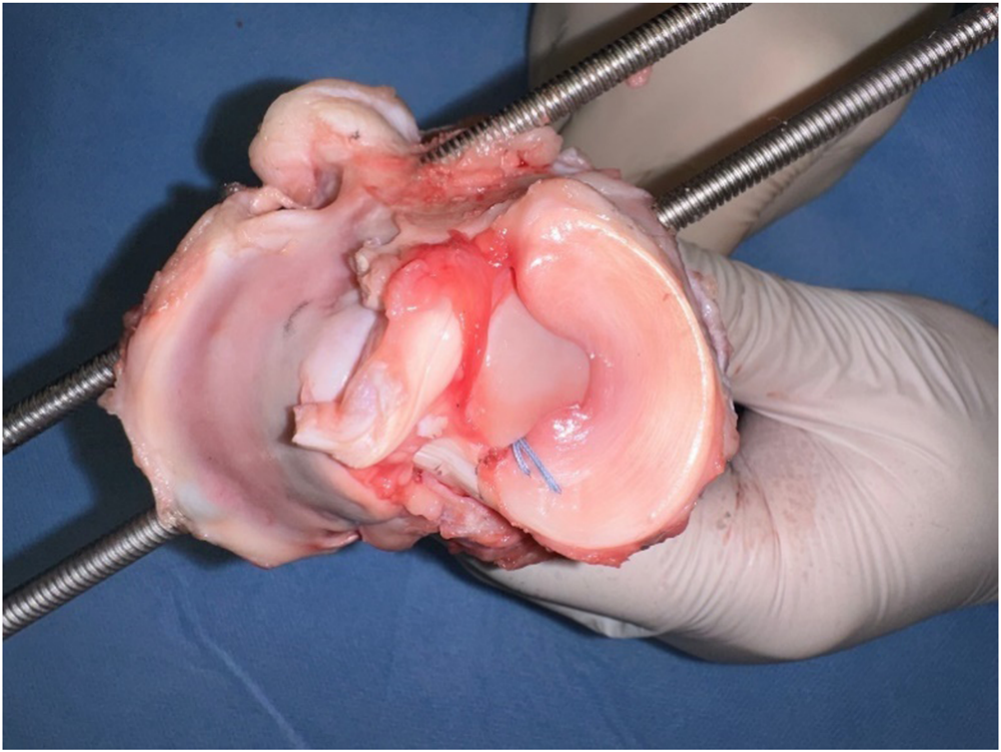
The meniscus was fixed at the posterior tibial plateau with anatomical position of the medial meniscus posterior root.

The meniscus was fixed at the posterior tibial plateau with anatomical position of the MMPR (Figure 3).

### Biomechanical testing

To apply load to the sutured menisci and test the desired biomechanical parameters, the tibia had to be secured in the testing machine. For this purpose, two 5 mm Steinmann pins were drilled parallel to each other at a distance of 2 cm from medial to lateral through the tibial bone. One pin was placed as far anteriorly towards the tibial tuberosity as possible, and the second pin was placed dorsally from this. Subsequently, metal plates with custom‐made hole devices were slid over the pins on both sides. These metal plates served as the superior connection for integration into the testing machine.

**Figure 4:**
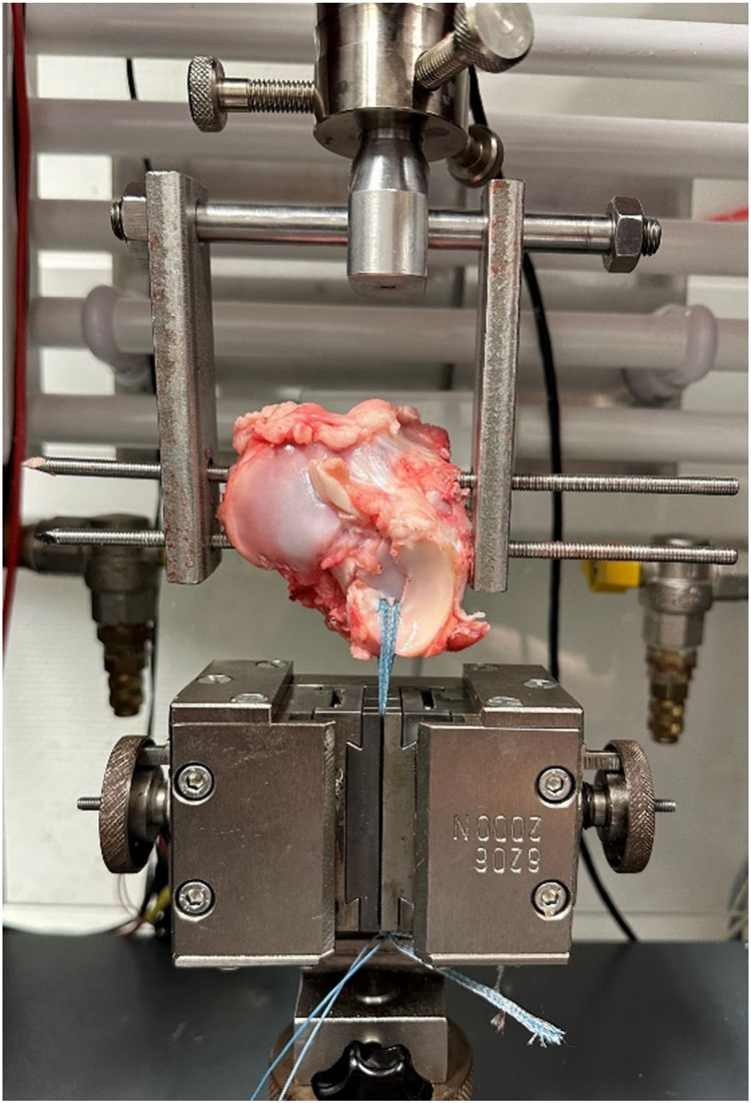
Biomechanical testing set‐up. The tibia was fixed with two 5 mm Steinmann Pins. The upper suspension pulls the tibia upwards while the 2 mm FiberTapes hold the meniscus in its position. The force vector is perpendicular to the repaired medial meniscus posterior root.

The inferior fixation in the testing machine was achieved using two grooved clamping boards. Two 2 mm FiberTapes (Arthrex, Naples, USA) were used to transmit the tensile force to the refixed MMPR. One 2‐mm FiberTape was looped between the two suture threads around the free meniscal surface, and the second one was placed medial to the medial thread. This allowed the MMPR to be held in place while the tibia was pulled superiorly, either repetitively or with maximum force, depending on the setting. This created a force vector that was precisely perpendicular to the MMPR (Figure 4). The two 2‐mm FiberTapes were clamped between the clamping boards in each trial so that their free span above the edge of the clamping boards was 30 mm.

Through this clamping device, the refixed MMPR was loaded by a force vector at a 90° orientation to the tibial shaft axis. This loading represents one of the strongest loads the root must withstand [3, 15].

Overall, by fixing the construct in the testing machine using this method, a three‐point fixation was achieved (two pins representing two points and the fixation sutures at the MMPR representing the third point), which securely and rigidly clamped the construct, minimising the risk of distorted elongation values.

All tests were then conducted at room temperature using the Zwick/Roell Z010 materials testing machine (Zwick/Roell, Ulm, Germany) with a 10‐kN load cell. testXpert II V3.1 was used as testing software.

The biomechanical tests were performed in consecutive sections. First, the specimens were installed in the testing machine as described above. Before starting the following protocols, force was set to 0 N. A cyclic preconditioning was performed with 20 loading cycles at a force interval of 1–5 N at a cycle speed at 200 mm/min. This is done to properly align the specimens. Followed by cyclic testing with 1000 cycles at a force interval of 5–20 N, similar to other protocols that have been used in biomechanical studies on porcine knees for repair techniques of meniscal root tears [11, 52]. This force application corresponds to the typical forces acting on the posterior horn of medial meniscus during knee flexion from 30° to 90° [3, 15]. The cycle speed was set at 200 mm/min. Finally, the force intensity was increased until failure. The failure mechanisms were documented.

To compare the individual fixation methods, the following data were collected and compared: elongation in mm during the 1000 cyclic loadings, stiffness in N/mm, yield load in N and maximum load to failure in N.

The failure mechanisms were observed and included: pullout of the lateral suture material through the meniscus, pullout of the medial and lateral suture material through the meniscus, failure of the tibial fixation (bypassing of the suture material at the IS or opening of the knots over the EB), pullout of the suture material through the lateral meniscus followed by failure of the tibial fixation, and pullout of the lateral suture material through the meniscus followed by failure of the suspension.

### Statistical analysis

We performed a sample size calculation based on the data of Robinson et al. [50] before testing. With an effect size of 1.25, a significance level of 0.05 and a power of 0.80 the sample size calculation was 10 per group (40 specimens at all). After the tests, a statistical analysis was conducted using SigmaPlot 12.5 software.

All variables were assessed for normal distribution using the Shapiro–Wilk test. To examine differences in variables between the individual biomechanical test groups, a one‐way analysis of variance (ANOVA) was performed for the yield load and maximum load to failure, followed by pairwise multiple comparison methods using the Holm‐Sidak method. Additionally, a Kruskal–Wallis one‐way analysis of variance on ranks (ANOVA on Ranks) was conducted for elongation and stiffness, followed by pairwise multiple comparison methods using the Tukey test.

The significance level was set at *p* ≤ 0.05. Post‐hoc analyses of our data (with G*Power) revealed an effect size of 1.81 and a power of 0.94 for maximum‐load‐to failure and an effect size of 1.7 and a power of 0.91 for yield load.

## RESULTS

### Elongation during cyclic loading

During all tests, there was no failure of the test setup. The median value of elongation for each of the four test groups, the minimum and maximum values of elongation, and significant differences between two test groups are presented in Table 1.

**Table 1:** Elongation during cyclic loading with 1000 cycles.

Group (method of fixation)	Number (*n*)	Median (mm)	Min. (mm)	Max. (mm)
Group 1 (IS and ST)	10	1.6	1.4	1.9
Group 2 (IS and FW)	10	1.4^a^ ^,^ ^b^	0.9	2.0
Group 3 (EB and ST)	10	1.9	1.3	2.9
Group 4 (EB and FW)	10	1.9	1.4	2.8

*Note*: Data are shown as median values with minimal and maximal elongation in mm.

Abbreviations: EB, Endobutton; FW, FiberWire; IS, interference screw; ST, SutureTape.

^a^ Group 2 showed significantly less elongation compared to Group 3 (*p* < 0.05).

^b^ Group 2 showed significantly less elongation compared to Group 4 (*p* < 0.05).

**Figure 5:**
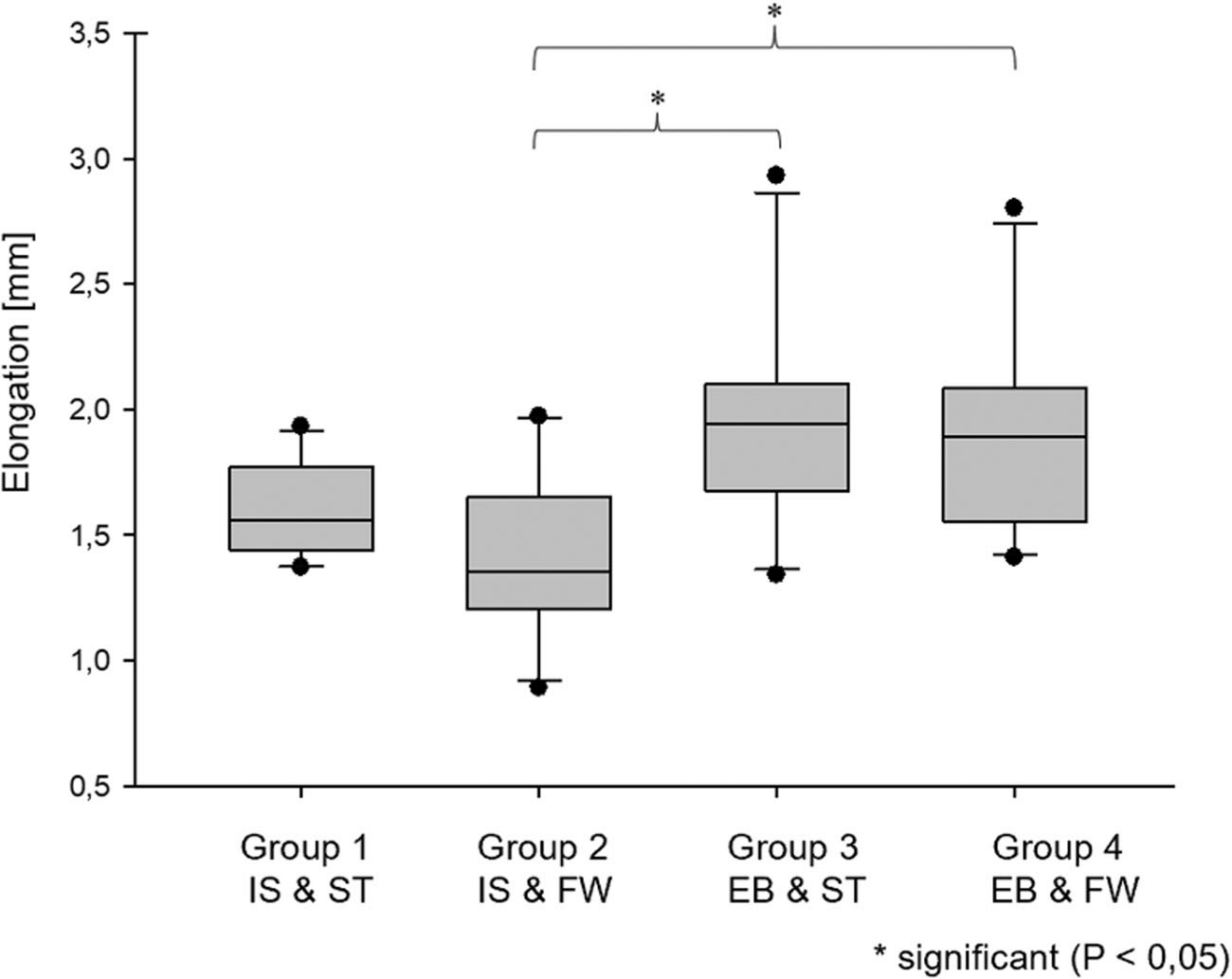
Elongation during cyclic loading (median values with 25th and 75th percentiles). Group 2 showed significantly less elongation compared to Groups 3 and 4 (*p* < 0.05) after 1000 cycles. EB, Endobutton; FW, FiberWire; IS, interference screw; ST, SutureTape.

Group 2 (IS and FW) showed a significantly lower elongation than Group 3 (EB and ST) and Group 4 (EB and FW) (*p* < 0.05). No significant differences were found between Group 1 (IS and ST) and Group 3 (EB and ST), as well as Group 4 (EB and FW). Likewise, no significant differences were found between Group 3 (EB and ST) and Group 4 (EB and FW), as well as Group 1 (IS and ST) and Group 2 (IS and FW) (*p* > 0.05). The construct of EB and ST (Group 3) showed the most elongation after 1000 cycles, closely followed by EB and FW (Group 4). The two groups with IS as tibial fixation (Groups 1 and 2) showed the least elongation (Figure 5).

#### Maximum load to failure and mode of failure

The average maximum load to failure of the respective four test groups, their standard deviations, minimum and maximum values, and the corresponding *p*‐values, in case of a significant difference between two groups, are listed in Table 2.

**Table 2:** Maximum load to failure.

Group (method of fixation)	Number (*n*)	Mean (N)	Standard deviation (N)	Min. (N)	Max. (N)
Group 1 (IS and ST)	10	428.8	111.8	306.0	677.6
Group 2 (IS and FW)	10	253.5^a^ ^,^ ^b^	132.0	50.7	488.0
Group 3 (EB and ST)	10	379.7	133.4	155.7	556.4
Group 4 (EB and FW)	10	405.2	89.9	268.8	553.0

*Note*: Data are shown as mean values with standard deviation and minimal and maximal load to failure in N*.*

Abbreviations: EB, Endobutton; FW, FiberWire; IS, interference screw; ST, SutureTape.

^a^ Group 2 showed significantly less maximum load to failure compared to Group 1 (*p* = 0.012).

^b^ Group 2 showed significantly less maximum load to failure compared to Group 4 (*p* = 0.034).

**Figure 6:**
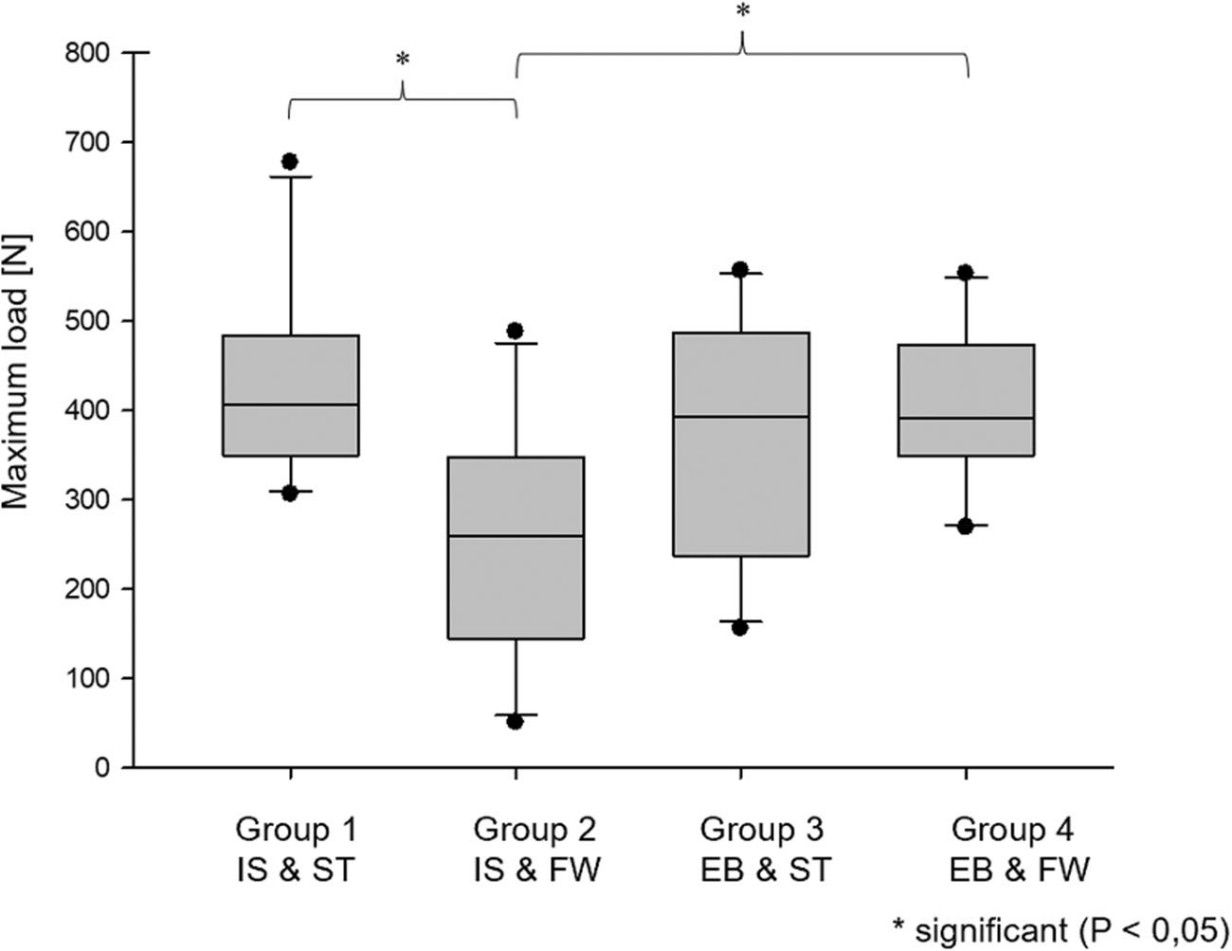
Maximum load to failure (mean values with standard deviations). Group 2 showed a significantly less maximum load compared to Group 1 (*p* = 0.012) and Group 4 (*p* = 0.034). EB, Endobutton; FW, FiberWire; IS, interference screw; ST, SutureTape.

The combination of IS and ST (Group 1) showed the highest maximum load to failure. Following that were EB and FW (Group 4) and EB and ST (Group 3). The construct of IS and FW (Group 2) exhibited the lowest load to failure. A statistically significant difference was observed between Group 1 (IS and ST) and Group 2 (IS and FW), as well as between Group 4 (EB and FW) and Group 2 (IS and FW) (*p* < 0.05). No further significant differences were found between the other groups (*p* > 0.05) (Figure 6).

**Figure 7:**
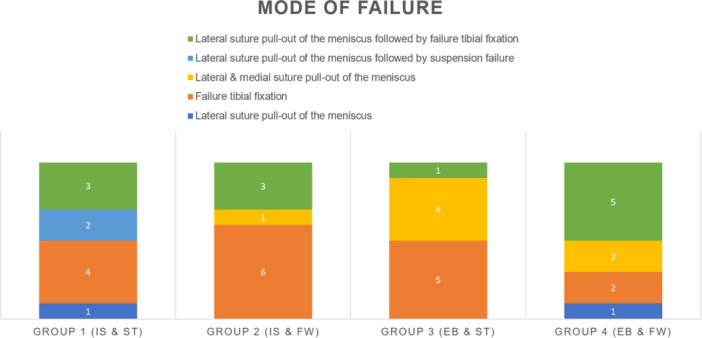
Mode of failure. The most common mode of failure was failure of tibial fixation (*n* = 17 of 40), followed by lateral suture pull‐out of the meniscus followed by failure of tibial fixation (*n* = 12 of 40). Medial and lateral suture pull‐out of the meniscus occurred at seven times (*n*= 7 of 40). Less common mode of failure were lateral suture pull‐out of the meniscus (*n* = 2 of 40) and lateral suture pull‐out of the meniscus followed by suspension failure (*n* = 2 of 40).

The most common failure mechanism overall was tibial fixation failure (*n* = 17 out of 40). Frequently seen subsequently was the pull‐out of the lateral suture through the meniscus followed by tibial fixation failure (*n* = 12 out of 40), pull‐out of both medial and lateral sutures through the meniscus (*n* = 7 out of 40), and then pull‐out of the lateral suture through the meniscus (*n* = 2 out of 40), as well as pull‐out of the lateral suture through the meniscus followed by suspension failure (*n* = 2 out of 40; Figure 7).

#### Yield load

The average yield load of the respective four test groups, their standard deviations, minimum and maximum values, and the corresponding *p*‐values, in case of a significant difference between two groups, are also listed in Table 3.

**Table 3:** Yield load.

Group (method of fixation)	Number (*n*)	Mean (N)	Standard deviation (N)	Min. (N)	Max. (N)
Group 1 (IS and ST)	10	320.6	105.0	217.0	520.0
Group 2 (IS and FW)	10	195.0^a^	114.9	33.0	345.0
Group 3 (EB and ST)	10	316.8	147.4	130.0	515.0
Group 4 (EB and FW)	10	362.3	83.9	256.0	548.0

*Note*: Data are shown as mean values with standard deviation and minimal and maximal yield load in N*.*

Abbreviations: EB, Endobutton; FW, FiberWire; IS, interference screw; ST, SutureTape.

^a^ Group 2 showed significantly less yield load compared to Group 4 (*p* = 0.015).

**Figure 8:**
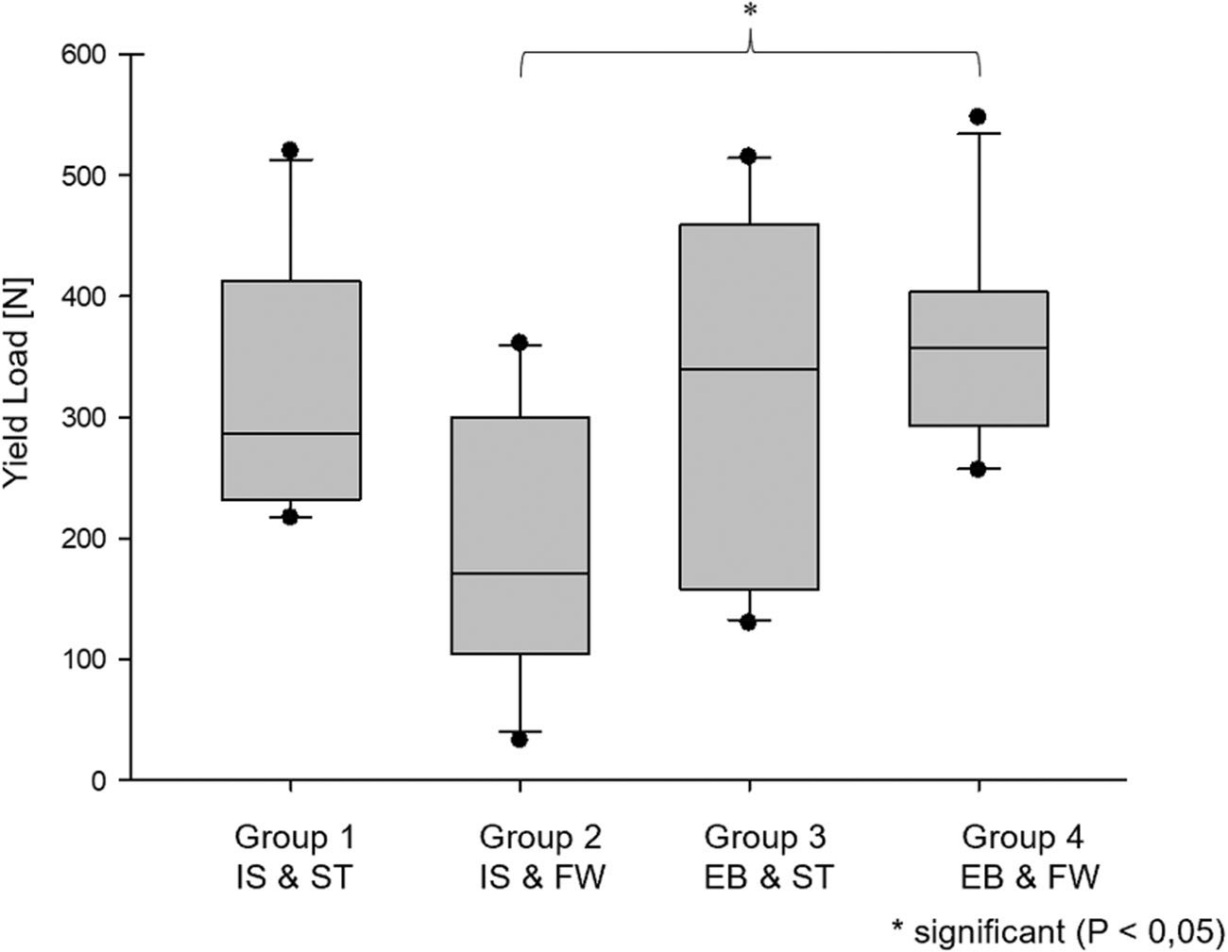
Yield load (mean values with standard deviations). Group 2 showed a significantly less yield load compared to Group 4 (*p* = 0.015). EB, Endobutton; FW, FiberWire; IS, interference screw; ST, SutureTape.

Group 4 (EB and FW) showed the highest yield load, with a significant difference compared to Group 2 (IS and FW) (*p* < 0.05). The second highest yield load was observed in Group 1 (IS and ST), followed by Group 3 (EB and ST), and finally Group 2 (IS and FW) (Figure 8). However, there were no further significant differences observed here (*p* > 0.05).

#### Stiffness

The results for stiffness are shown in Table 4.

**Table 4:** Stiffness.

Group (method of fixation)	Number (*n*)	Median (N/mm)	Min. (N/mm)	Max. (N/mm)
Group 1 (IS and ST)	10	51.2^a^ ^,^ ^b^	41.4	61.0
Group 2 (IS and FW)	10	48.5^c^ ^,^ ^d^	42.5	66.2
Group 3 (EB and ST)	10	35.6	29.3	54.6
Group 4 (EB and FW)	10	35.6	31.7	40.2

*Note*: Data are shown as median values with minimal and maximal stiffness in N/mm.

Abbreviations: EB, Endobutton; FW, FiberWire; IS, interference screw; ST, SutureTape.

^a^ Group 1 showed significantly higher stiffness compared to Group 3 (*p* < 0.05).

^b^ Group 1 showed significantly higher stiffness compared to Group 4 (*p* < 0.05).

^c^ Group 2 showed significantly higher stiffness compared to Group 3 (*p* < 0.05).

^d^ Group 2 showed significantly higher stiffness compared to Group 4 (*p* < 0.05).

**Figure 9:**
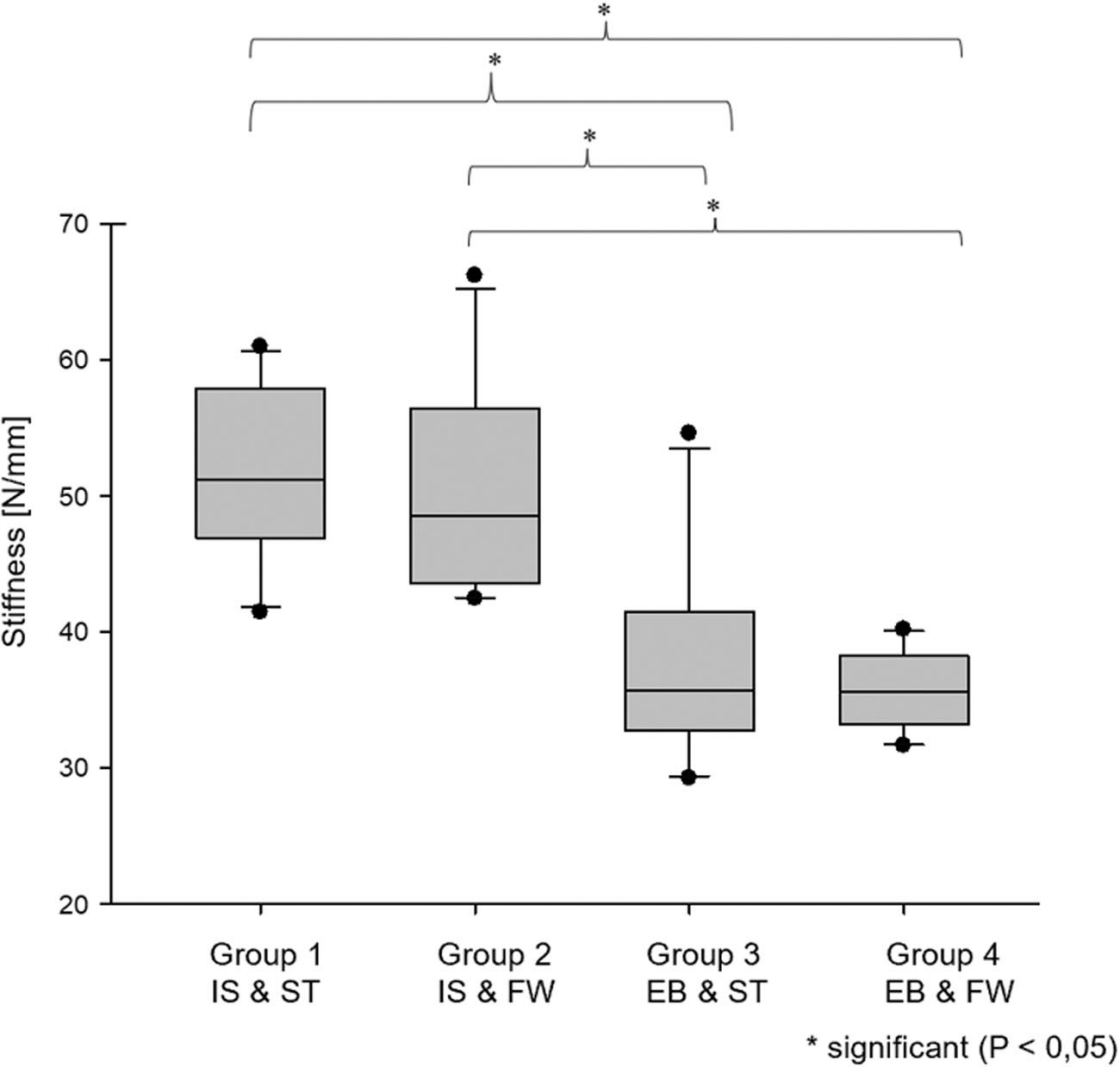
Stiffness (median values with 25th and 75th percentiles). Group 1 showed a significantly higher stiffness compared to Groups 3 and 4 (*p* < 0.05). Group 2 showed a significantly higher stiffness compared to Groups 3 and 4 (*p* < 0.05). EB, Endobutton; FW, FiberWire; IS, interference screw; ST, SutureTape.

Group 1 (IS and ST) exhibited a significantly higher stiffness compared to Group 4 (EB and FW) and Group 3 (EB and ST) (*p* < 0.05). Similarly, Group 2 (IS and FW) showed greater stiffness than Group 4 (EB and FW) and Group 3 (EB and ST) (*p* < 0.05). The combination of IS and ST (Group 1) emerged as the stiffest overall construct followed by Group 2 (IS and FW), Group 3 (EB and ST) and Group 4 (EB and FW) (Figure 9). There were no significant differences observed between groups with the same tibial fixation (*p* > 0.05).

## DISCUSSION

In this study, transtibial fixation of pull‐out suture using IS resulted in reduced elongation and increased stiffness, compared to fixation using EB. These biomechanical advantages are likely due to the IS anchoring the suture deeper within the bone tunnel, minimising the free suture length and thereby reducing micromotion—an important factor for root healing [5, 55]. In contrast, EB fixation may allow soft tissue interposition between the button and cortex, potentially compromising stability. Additionally, the method using IS is technically easy and tension of the sutures can be adjusted appropriately [46].

Our results also support the hypothesis that the biomechanical behaviour of the construct depends on the specific combination of fixation device and suture material. While the IS/ST (Group 1) combination demonstrated superior biomechanical performance, the IS/FW (Group 2) combination was significantly weaker in terms of maximum load to failure and yield load. This may be attributed to the broader contact surface of ST, which allows for more even force distribution, reduces stress concentration and thus increase the maximum load to failure [11, 41]. Despite its higher rigidity and lower elongation [41], FW's thinner profile may contribute to earlier tissue cut‐out or construct failure under load. These findings suggest that the choice of suture material should consider both load‐bearing capacity and interaction with the chosen fixation method.

From a clinical perspective, it is essential that any repair construct can withstand physiological loads encountered during early rehabilitation. For instance, under 500 N axial load and 90° of knee flexion, approximately 60.1 N is transmitted through the medial meniscus [56]. Additionally, 100 N has been identified as a critical threshold during postoperative regimen [51]. All constructs in this study exceeded these thresholds, indicating their potential suitability for postoperative rehabilitation. Notably, Robinson et al. [50] demonstrated sufficient primary stability using multiple fixation techniques but did not include IS in their study. Our results suggest that combining IS with ST may offer an even more robust alternative for early‐phase stability.

The materials and techniques examined here are already in clinical use and have shown favourable clinical outcomes in humans [7, 17, 18, 34]. However, failure at the meniscus‐suture interface remains a commonly reported issue in MMPRT repair and is a leading cause of displacement or elongation under cyclic loading [5]. Several techniques have been proposed to address this, including the two simple sutures (TSS) technique, modified Mason‐Allen suture (MMA), single double‐locking loop (S‐DLL), double double‐locking loop (D‐DLL) and modified Kessler suture (MK) [30, 33, 43, 53]. In this study, we selected the TSS technique, which has previously demonstrated lower displacement than S‐DLL or D‐DLL configurations [32] and provides sufficient resistance under physiologic loads. While the MK technique may offer higher failure loads in vitro, none of the current suture methods can fully replicate the biomechanical properties of the native meniscus root [29]. Clinical studies have shown that TSS performs reliably under functional loading conditions [17].

Polyfilament structured sutures such as tapes and wires are increasingly favoured due to their high pullout resistance and low elongation [4, 11, 41, 45, 50]. Their efficacy in clinical applications has been documented in multiple studies [17, 18, 34], making them suitable for use in biomechanically demanding repairs.

A displacement greater than 3 mm is typically considered failure in MMPRT repair constructs [45], and this metric was used as a reference in our analysis in terms of elongation. A significant part of elongation occurs within the first 20 cycles of loading [51], which underlines the importance of optimising the fixation construct to minimise early elongation. Potential sources of displacement include elongation at the suture‐meniscus interface, within the suture material itself, and at the tibial fixation site [5].

Due to physiological meniscal mobility during knee motion, excessive construct rigidity may also lead to early pull‐out from the meniscus [27]. This may explain the limited healing capacity observed on second‐look arthroscopies [53].

One specific failure mode observed in IS groups involved the suture material bypassing the IS under low load. This occurred more frequently with FW (*n* = 6) than with ST (*n* = 4), likely due to differences in surface geometry. Additional knotting after tunnel exit may help reduce this risk, but further comparative testing is warranted.

This study has several limitations. First, a native control group representing the primary stability of the porcine meniscus was not included. Mitchell et al. [42] attempted such an evaluation in human cadaveric knees but reported construct failure prior to meniscal rupture, a limitation we also encountered in our preliminary testing. The primary fixation strength of the native meniscus was substantially higher than that of any repaired MMPRT construct, leading us to discontinue further testing with native specimens.

Although cyclic loading was used to mimic repetitive stress, the study ultimately captures only time‐zero mechanics. Extending testing to 1500 or 3000 cycles in future studies may reveal later‐stage failure patterns, although most elongation is believed to occur within the initial 20 cycles [51].

Furthermore, the IS was inserted under tension to ensure proper adaptation of the MMPRT, but excessive tension may have increased the risk of suture pull‐out [15]. Due to the transected femur, precise control of flexion angle and suture tensioning was not feasible. Future studies should incorporate tensioning devices as described by Hiranaka et al. [18].

The 2 mm FiberTapes used to apply tensile load could themselves elongate or cut into the meniscus. They were intentionally chosen due to their broader contact area, which may reduce focal stress [11] and avoid pull‐out of the securing stitches, which was not observed in any of our samples. While there were only two cases of suspension failure, both occurred after meniscal pullout had already initiated. Because the elongation characteristics of the FiberTapes were identical across all test groups, their influence on intergroup comparisons is considered negligible. For absolute intra‐group elongation measurements, their inherent elongation would need to be subtracted, though this would be of limited relevance due to the use of porcine tissue.

The use of porcine knees for in vitro studies is common [10, 11, 50, 51, 61]. Although the validity of this study is limited by this, we can test biomechanical fundamentals on the porcine model, as all comparison groups meet the same conditions. It is important to note that porcine menisci have more consistent mechanical properties compared to older human cadavers and are anatomically and functionally comparable to menisci in young adults [11]. While the ovine model offers closer anatomical resemblance to the human meniscus [58], anatomical similarity alone does not suffice as a selection criterion of animal models. Further investigations are required to determine the biomechanical and structural comparability between human and sheep menisci [58].

## CONCLUSION

In this porcine model, transtibial pull‐out repair of MMPR tears using IS demonstrated superior primary biomechanical stability compared to EB fixation. Constructs with IS showed reduced elongation and increased stiffness under load. However, to confirm the clinical relevance of these findings, further investigations in clinical settings are necessary.

## AUTHOR CONTRIBUTIONS

Timo Spannagel and Stephan Reppenhagen formulated the hypotheses and conducted the biomechanical work. Isabell Biermann and Bastian Schäfer instructed Timo Spannagel and Stephan Reppenhagen in the use of the testing machine. Timo Spannagel was instructed from Philip Anderson in the statistical analysis. Timo Spannagel then performed the statistical analysis. Timo Spannagel was responsible for preparing the statistical analysis and compiling the results in the scientific context. Stephan Reppenhagen and Timo Spannagel co‐wrote the final article together. All authors read and approved the final manuscript.

## CONFLICT OF INTEREST STATEMENT

The authors declare no conflicts of interest.

## ETHICS STATEMENT

The porcine knees used in this study were sourced from a local abattoir. The pigs were not specifically slaughtered for this study; rather, they were slaughtered by the abattoir according to standard procedures. The porcine knees that were not suitable for other purposes were utilised for this study.

## Data Availability

The data sets used and analysed during the current study are available from the corresponding author on reasonable request.
